# Structural insights into conformational switching in latency-associated peptide between transforming growth factor β-1 bound and unbound states

**DOI:** 10.1107/S205225251901707X

**Published:** 2020-02-06

**Authors:** Timothy R. Stachowski, Mary E. Snell, Edward H. Snell

**Affiliations:** a Hauptman–Woodward Medical Research Institute, 700 Ellicott Street, Buffalo, NY 14203, USA; b Cell Stress Biology, Roswell Park Comprehensive Cancer Center, 665 Elm Street, Buffalo, NY 14203, USA; c Materials Design and Innovation, State University of New York at Buffalo, 700 Ellicott Street, Buffalo, NY 14203, USA

**Keywords:** latency-associated peptide, transforming growth factor β-1, growth factors, structural biology, TGFβ-1

## Abstract

Transforming growth factor β-1 (TGFβ-1) is a secreted signalling protein that directs many cellular processes and is an attractive target for the treatment of several diseases. The primary endogenous activity regulatory mechanism for TGFβ-1 is sequestration by its pro-peptide, latency-associated peptide (LAP), which sterically prohibits receptor binding by caging TGFβ-1. As such, recombinant LAP is promising as a protein-based therapeutic for modulating TGFβ-1 activity; however, the mechanism of binding is incompletely understood. Comparison of the crystal structure of unbound LAP (solved here to 3.5 Å resolution) with that of the bound complex shows that LAP is in a more open and extended conformation when unbound to TGFβ-1. Analysis suggests a mechanism of binding TGFβ-1 through a large-scale conformational change that includes contraction of the inter-monomer interface and caging by the ‘straight-jacket’ domain that may occur in partnership through a loop-to-helix transition in the core jelly-roll fold. This conformational change does not appear to include a repositioning of the integrin-binding motif as previously proposed. X-ray scattering-based modelling supports this mechanism and reveals possible orientations and ensembles in solution. Although native LAP is heavily glycosylated, solution scattering experiments show that the overall folding and flexibility of unbound LAP are not influenced by glycan modification. The combination of crystallography, solution scattering and biochemical experiments reported here provide insight into the mechanism of LAP sequestration of TGFβ-1 that is of fundamental importance for therapeutic development.

## Abstract 2

Analysis of crystallographic, solution scattering and biochemical experiments suggests a mechanism for the sequestration of transforming growth factor β-1 by latency-associated peptide (LAP) that includes a structural change in LAP from an open to a closed conformation. This mechanism contains several potential regulatory features that are exploitable for therapeutic development.

## 1. Introduction   

Transforming growth factor β-1 (TGFβ-1) is a potent growth-regulatory protein that has garnered much attention for its key roles in metazoan development (Wu & Hill, 2009 ▸), cell proliferation (Bierie & Moses, 2006 ▸) and immunity (Sanjabi *et al.*, 2017 ▸). Owing to its ubiquity in biological processes, cells maintain a delicate balance of TGFβ-1 expression and activity that, when disrupted, contributes to disease states (Janssens *et al.*, 2003 ▸; Akhurst & Hata, 2012 ▸). The fundamental mechanism of activity regulation is through co-secretion with its pro-domain, latency-associated peptide (LAP). LAP is a disulfide-linked dimer that noncovalently cages TGFβ-1 and blocks it from binding receptors on cell surfaces and initiating signalling pathways. This complex is known as latent TGFβ-1 (LTGFβ-1). Owing to its function in sequestering TGFβ-1, LAP is a pivotal mediator between TGFβ-1 signalling effects and cellular stimuli. LAP is the target of viral glycoside hydrolases (Carlson *et al.*, 2010 ▸), proteases (Sato & Rifkin, 1989 ▸) and cell-adhesion proteins (Dong *et al.*, 2017 ▸; Ribeiro *et al.*, 1999 ▸; Munger *et al.*, 1998 ▸) that induce large-scale conformational changes in LAP to release TGFβ-1 (McMahon *et al.*, 1996 ▸). The dissociation of TGFβ-1 from LAP is referred to as TGFβ-1 activation.

Although it is known that LAP undergoes a large conformational change upon latent complex formation and dissociation, the underlying structural mechanism is incomplete. Circular-dichroism (CD) studies observed a drastic change in the secondary structure of LAP from a mixed composition in the bound state to a composition that is mainly β-sheet in the apo state (McMahon *et al.*, 1996 ▸). A comparison of CD results with the structure of the LAP domain in the LTGFβ-1 crystal structure suggested that binding of LAP to TGFβ-1 induces the formation of the α1 helix in the N-terminal straight-jacket domain. Solution studies observed that apo LAP is highly flexible and this is most likely to be because the straight-jacket is extended and unstructured in the absence of TGFβ-1 (Stachowski *et al.*, 2019 ▸). Although biochemical studies demonstrated that the straight-jacket domain is important in assembling the latent complex and maintaining bound TGFβ-1 (Walton *et al.*, 2010 ▸), the LTGFβ-1 crystal structure revealed that several residues in the fastener region, which also form an interface with TGFβ-1, are essential for latent complex formation (Shi *et al.*, 2011 ▸). Despite these observations, a cohesive sequence of conformational changes in LAP during TGFβ-1 binding remains incomplete.

Since the straight-jacket domain also contains binding motifs for several proteases, the exhibited structural plasticity of this domain between TGFβ-1 binding states is thought to be advantageous as a regulatory mechanism in which protein binding prohibits the reassociation of LAP with TGFβ-1 following release (Schultz-Cherry *et al.*, 1995 ▸; Ribeiro *et al.*, 1999 ▸; Sato & Rifkin, 1989 ▸). Similarly, although integrins (cell-adhesion proteins) bind LAP to release TGFβ-1, biochemical studies observed that integrins exhibited increased binding to apo LAP compared with LAP complexed with TGFβ-1. This was hypothesized to be owing to a repositioning of the integrin-binding RGD motif in LAP between TGFβ-1 binding states that would make it more accessible in the unbound form (Munger *et al.*, 1998 ▸). Crystal structures of LTGFβ-1 (Shi *et al.*, 2011 ▸) and of LTGFβ-1 in complex with integrin (Dong *et al.*, 2017 ▸) support this, showing that the RGD-containing loop is highly variable and undergoes a large structural change upon integrin binding (Dong *et al.*, 2017 ▸; Zhao *et al.*, 2017 ▸). These studies show that conformational changes in LAP play a direct functional role in the formation and activation of the latent complex. However, an atomic structure showing the position of the RGD motif and the straight-jacket in LAP in the unbound state has remained elusive, most probably owing to extensive conformational heterogeneity and glycosylation.

The LAP dimer is heavily glycosylated with six N-linked complex-type glycans on the arm (Brunner *et al.*, 1992 ▸), but the importance of glycosylation in LTGFβ-1 is also unclear. Although first identified by Miyazono and coworkers in 1988 (Miyazono *et al.*, 1988 ▸), how the glycans are chemically modified remains contentious (Barnes *et al.*, 2012 ▸). For proper latent complex processing, the presence of glycosylation seems to be necessary in only certain cell types (Brunner *et al.*, 1992 ▸; Shi *et al.*, 2011 ▸; Munger *et al.*, 1998 ▸; McMahon *et al.*, 1996 ▸). On the other hand, glycan targeting by treatment with endoglycosidase F, sialidase (Miyazono & Heldin, 1989 ▸) or neuro­aminidase (Carlson *et al.*, 2010 ▸) can result in the release of TGFβ-1 from LAP. Since glycosylation influences protein folding (Mitra *et al.*, 2006 ▸), stability (Wang *et al.*, 1996 ▸) and protein–protein interactions such as proteolysis (Russell *et al.*, 2009 ▸), there is considerable pharmaceutical interest in modifying glycosylation to improve therapeutic efficacy (Walsh & Jefferis, 2006 ▸). However, because glycosylation is thought to improve protein chemical stability through reducing conformational dynamics (Lee *et al.*, 2015 ▸; Bager *et al.*, 2013 ▸), determining whether glycosylation contributes to the folding of LAP is fundamental to understanding its biological activity.

Detailing the structural differences in LAP between TGFβ-1 binding states is crucial for understanding how to use LAP as a tool to modulate TGFβ-1 activity, where there is a large interest in the development of targeted antibodies for LAP (Gabriely *et al.*, 2017 ▸; da Cunha *et al.*, 2015 ▸) and treatments with recombinant LAP (Wilkinson *et al.*, 2000 ▸; Nakamura-Wakatsuki *et al.*, 2012 ▸; Zhang *et al.*, 2003 ▸; Ali *et al.*, 2008 ▸; Böttinger *et al.*, 1996 ▸). Here, we report the crystal structure of human LAP in the apo state at a resolution of 3.5 Å. The straight-jacket domain could not be resolved in the crystal structure, which supports previous reports that it is conformationally dynamic (Stachowski *et al.*, 2019 ▸). Comparison of the apo and TGFβ-1 bound LAP structures revealed that that the globular arm domains are slightly rotated with respect to one another and indicates that residues adjacent to the disulfide-linked dimer interface function as a hinge. Together, this positions LAP in a more open conformation than in the bound structure, perhaps improving accessibility for TGFβ-1 binding. Morphing between bound and unbound LAP structures suggests that binding of TGFβ-1 by contracting the arm domains and wrapping by the straight-jacket domain may occur in concert through a previously unidentified loop-to-helix transition in the core jelly-roll fold. This is supported by biochemical experiments showing that the formation of the helix is necessary for proper folding of LAP and TGFβ-1 into the latent complex. X-ray scattering-based modelling supports this mechanism and reveals possible orientations and ensembles in solution. However, this large-scale conformational change in LAP during TGFβ-1 binding does not seem to include a repositioning of the integrin-binding motif as previously thought because the loop containing the motif is similarly positioned in the apo and bound structures. Lastly, solution scattering experiments with different LAP glycoforms show that the overall folding and flexibility of unbound LAP are not influenced by glycosylation.

## 2. Materials and methods   

### 2.1. Macromolecule production   

The LAP domain (residues 30–278) of human LTGFβ-1 (UniProtKB accession No. P01137) was expressed similarly and purified as described previously (Stachowski *et al.*, 2019 ▸). Briefly, a plasmid containing LTGFβ-1 was obtained from Addgene. The TGFβ-1 domain was removed using a single PCR reaction. The primers are shown in Supplementary Table S1. An R278A mutation was produced to prohibit endogenous proteolysis of the C-terminal His_6_ tag and a C4S mutation was included to improve expression. The recombinant protein was expressed in Expi-HEK293F cells grown in suspension while shaking with Expi293 medium at 37°C and 8% CO_2_ (Thermo Fisher). 4–6 h post-transfection, 5 µ*M* kifunensine (Tocris) was added to homogenize N-linked glycosylation to the high-mannose branching type and to sensitize the glycosides to subsequent enzymatic digestion. Expression continued for a total of 48–72 h before harvesting. The medium containing the secreted protein was separated from the cells by centrifugation and filtration. The clarified medium was concentrated tenfold by tangential flow filtration and diluted tenfold in Tris-buffered saline pH 8.0. The protein was purified with Ni–NTA (Marvelgent). LAP expressed in the presence of kifunensine was enzymatically deglycosylated with Endoglycosidase H (EndoH; New England Biolabs). Samples were further purified using size-exclusion chromatography (GE Healthcare) and exchanged into the crystallization buffer. Macromolecule-production information is summarized in Supplementary Table S1.

### 2.2. Crystallization   

LAP expressed in the presence of kifunensine was initially screened for crystallization using a high-throughput microbatch-under-oil method at the Hauptman–Woodward Institute High Throughput Crystallization Screening Center (Luft *et al.*, 2003 ▸). Crystal hits were optimized and grown by mixing 1 µl concentrated protein solution with 1 µl reservoir solution at room temperature. Before cryocooling in liquid nitrogen, five rounds of increasing the PEG 400 concentration (2 min for each increase of 5%) were carried out in reservoir solution that was also supplemented with 5% PEG 3350. Crystallization information is summarized in Supplementary Table S2.

### 2.3. Data collection and processing   

Diffraction data were collected from a single crystal on beamline 17-ID (IMCA-CAT) at the Advanced Photon Source (APS), Argonne National Laboratory (ANL). The data were integrated with *MOSFLM* (Battye *et al.*, 2011 ▸; Powell, 1999 ▸) and scaling was performed with *AIMLESS* (Evans & Murshudov, 2013 ▸). Detailed statistics of the data collection and processing are shown in Table 1 ▸.

### 2.4. Structure solution and refinement   

The structure was determined by molecular replacement by iteratively rebuilding the structure of inactivatable human LTGFβ-1 (PDB entry 5vqp; Zhao *et al.*, 2017 ▸) with *Rosetta* and improving the phase solutions with *Phaser*, as implemented in *MR-ROSETTA* (translation-function *Z*-score 15.4; DiMaio *et al.*, 2011 ▸). The structural model was built using *AutoBuild* (Terwilliger *et al.*, 2008 ▸) and manually in *Coot* (Emsley *et al.*, 2010 ▸), and was refined using *Phenix* (Liebschner *et al.*, 2019 ▸) and *Rosetta* (DiMaio *et al.*, 2013 ▸). Validation was carried out with *MolProbity* (Chen *et al.*, 2010 ▸). The coordinates were deposited as PDB entry 6p7j. Structure-refinement statistics are provided in Table 1 ▸. Alignments between apo LAP and LTGFβ-1 (PDB entry 3rjr; Shi *et al.*, 2011 ▸) were performed using the ‘align’ function in *PyMOL* (Schrödinger) and only residues modelled in the apo structure were included for comparison. Domain and secondary-structure naming conventions follow Shi *et al.* (2011 ▸). The inter-monomer angle was calculated using the ‘angle_between_domains’ tool in *PyMOL* (T. Holder, Schrödinger). Structural figures were prepared using *PyMOL*. Morph files to reveal the extent of the structural changes between PDB entries 3rjr and 6p7j were generated using *UCSF Chimera* (Pettersen *et al.*, 2004 ▸).

### 2.5. Assessing the role of the α3 helix in forming LTGFβ-1   

Proline mutants in LAP were assessed for their ability to properly fold the latent complex by measuring the amount of TGFβ-1 trafficked into the extracellular matrix. Mutations were introduced into full-length LTGFβ-1_C4S_ using the Q5 Site-Directed Mutagenesis Kit (NEB) and were confirmed by DNA sequencing. Wild-type and mutant LTGFβ-1 were produced by transient transfection in HEK-293T cells using Lipofectamine 2000 (Thermo Fisher). Briefly, cells were seeded at 6.0 × 10^5^ cells per well in a six-well plate. After 24 h, wild-type or mutant LTGFβ-1 DNA (4 µg) was combined with Lipofectamine and added directly to plated cells according to the manufacturer’s instructions. The cells were cultured in serum-free medium for 48 h at 37°C in 5% CO_2_. 48 h post-transfection, the culture medium was removed and assessed for TGFβ-1 by sandwich ELISA. Anti-TGFβ-1 antibody-coated plates were prepared according to the manufacturer’s instructions (R&D Systems, catalogue No. DY240). To detect TGFβ-1, TGFβ-1 was released from LAP (activated) by incubating the supernatants with 1 *N* HCl for 10 min at room temperature (Walton *et al.*, 2010 ▸). The reaction was neutral­ized with 1.2 *N* NaOH in 0.5 *M* HEPES and ELISA was performed according to the manufacturer’s instructions. Transfections were repeated three times, and each time the amount of TGFβ-1 was measured in triplicate. The amount of DNA received by cells was assumed to be equally variable across samples and replicates. Transfections were also performed with a construct of LAP alone (no TGFβ-1 domain) to ensure antibody specificity and an empty construct (mock) to ensure that the results were not influenced by endogenous TGFβ-1. Also, TGFβ-1 was measured before and after acid activation to ensure that the amounts observed reflected TGFβ-1 that was trafficked in the latent complex and not independently of LAP. Values are expressed as the mean ± the standard deviation. Statistical comparisons were performed with a Student’s *t*-test. A statistical difference was considered significant if *p < 0.05 or **p < 0.01.

### 2.6. SAXS data collection   

To mitigate radiation damage from radicals and solvated electrons, purified LAP protein was exchanged into PBS pH 7.4 containing 2% glycerol using a Zeba desalting column (Stachowski *et al.*, 2019 ▸; Thermo Fisher). LAP protein was concentrated to 1.4 mg ml^−1^ (*A*_280_) using a 30 000 Da molecular-weight cutoff centrifugal concentration device (Amicon). After concentration, samples were diluted 1:2 using flowthrough buffer to create a concentration series. Data sets were collected on the SIBYLS beamline 12.3.1 at the Advanced Light Source synchrotron-radiation facility. Momentum-transfer values were calculated as *q* = 4πsinθ/λ, where 2θ is the scattering angle and λ is the X-ray wavelength in Å. Data were recorded using a PILATUS 2M detector (Dectris). Error bars were estimated using the *GNOM* program from *ATSAS* (Svergun, 1992 ▸; Franke *et al.*, 2017 ▸). A volume of 25 µl of each sample was loaded into the sample chamber (Dyer *et al.*, 2014 ▸). The exposure time for each frame was 0.1 s and a total of 100 frames were collected for each sample, with the sample kept in a fixed position. Scattering from buffer samples was subtracted from the corresponding protein sample to generate the SAXS scattering profiles. Data-collection parameters are summarized in Supplementary Table S4.

### 2.7. SAXS data analysis   

Primary data analysis was conducted in *ATSAS* v.2.8.3 (Franke *et al.*, 2017 ▸). Prior to averaging, exposures were monitored for radiation damage by comparing the radius of gyration (*R*_g_) and analysing the total scattering shape using *CorMap* (Franke *et al.*, 2015 ▸). The *R*_g_ values reported were calculated from the Guinier region with ranges according to *q*_max_ × *R*_g_ = ∼1.3. Molecular weights were calculated from the volume of correlation (*V*_c_; Rambo & Tainer, 2013 ▸) and *D*_max_ values were calculated from the *P*(*r*) functions using *GNOM* in *ATSAS* (Svergun, 1992 ▸; Franke *et al.*, 2017 ▸). *P*(*r*) functions were normalized by the total area under the curve for clearer comparison between samples. Elongation factor (EF) ratios were calculated from the *P*(*r*) functions according to Putnam (2016 ▸) using a custom *Mathematica* script. All data sets were truncated to *q*_max_ = 0.25 Å^−1^ and missing residues in high-resolution structures were built with *MODELLER* via *UCSF Chimera* (Yang *et al.*, 2012 ▸) prior to comparison with high-resolution structures or rigid-body modelling. To compare the agreement between atomistic structures and SAXS data for LAP constructs, a scattering profile for the LAP domain from the LTGFβ-1 crystal structure (PDB entry 3rjr; Shi *et al.*, 2011 ▸) was generated using *CRYSOL* v.2.8.3 (Svergun *et al.*, 1995 ▸). Dimensionless Kratky plots were calculated in *RAW* (Hopkins *et al.*, 2017 ▸). χ^2^ values from comparisons between scattering curves were calculated using *DATCMP* (Franke *et al.*, 2017 ▸). The software employed for analysis is summarized in Supplementary Table S5 and experimental scattering characteristics are summarized in Supplementary Table S6.

### 2.8. Rigid-body modelling   

*CORAL* (v.1.1; Petoukhov *et al.*, 2012 ▸) was used to investigate the contributions of both the straight-jacket domain and the bowtie hinge conformations to the observed flexibility of apo LAP. The default settings without imposing symmetry were used for all calculations. The apo LAP (PDB entry 6p7j) crystal structure was used as the core model and the conformations of the missing residues 1–75, which mainly compose the straight-jacket domain, were sampled by *CORAL*. To simulate the disulfide bonds of the bowtie region while allowing movement between monomers, a contact condition restricting the maximum distance between C^α^ atoms to 6 Å was used for each of the two inter-monomer disulfides. Some 25 independent models were generated and clustered into conformationally related subfamilies using only the arm domains (owing to the extensive heterogeneity of the straight-jacket domain) with *DAMCLUST* (Petoukhov *et al.*, 2012 ▸). However, the reported χ^2^ values were calculated using the entire model. The two clusters that contained more than a single member are reported here. After superimposing a single chain from a rigid-body model onto a single chain of the bound LAP structure, the angle and translation (displacement) required to align the second pair of monomers was calculated with the ‘angle_between_domains’ tool in* PyMOL* (T. Holder, Schrödinger). The values from this procedure represent the total difference in rotation and displacement between the dimers.

### 2.9. Ensemble optimization   

The *Ensemble Optimization Method* (*EOM*), which determines an ensemble of structures that best explain the experimental SAXS data (v.2.2; Tria *et al.*, 2015 ▸), was used to determine whether an ensemble of models with varying rotations between monomers and straight-jacket conformations explained the scattering data better than an ensemble of only straight-jacket conformations. This was performed by comparing an *EOM* run with a pool of models in which the straight-jacket domain (amino acids 1–75) was built around rigid bodies of (i) three *CORAL* models with different inter-monomer rotations (−26, 47 and 84°), (ii) the apo LAP crystal structure (15°) and (iii) the bound LAP crystal structure (0°) (five core components in total) with an *EOM* run with a pool of models built only around the apo LAP crystal structure (one core component). Using this approach, pools containing 10 000 models for each component were generated in *RANCH* (part of *EOM*) with *P*2 symmetry, in which the straight-jacket domain was rebuilt in random conformations. The theoretical scattering of the resulting pool was calculated using *CRYSOL*. A genetic algorithm (*GAJOE*, from *EOM*) was used to select an ensemble of conformations from the random pool that best explained the SAXS data and was repeated 100 times, with the ensemble with the lowest discrepancy considered for analyses. This genetic algorithm protocol was repeated three times for each pool and averaged.

## 3. Results   

### 3.1. Overall structure and comparison of the LAP crystal structure in TGF-1 bound and unbound conformations   

**Figure 1:**
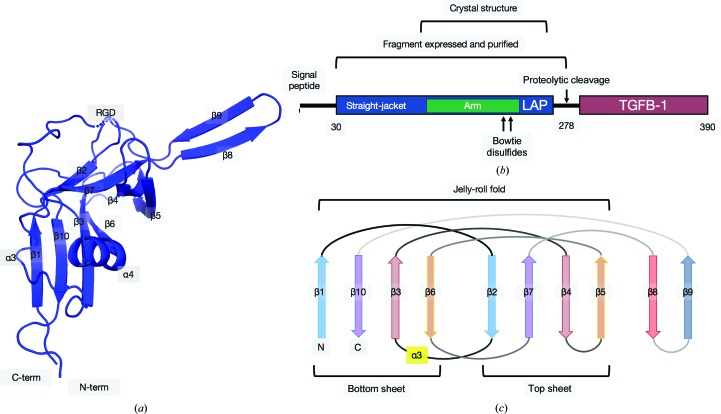
The architecture of the apo LAP crystal structure. (*a*) The asymmetric unit of the crystal structure of apo LAP shown as a cartoon model. Naming conventions follow those of Shi *et al.* (2011 ▸). Nonterminal missing regions are indicated by dashed lines. (*b*) A schematic of the LTGFβ-1 gene and the residues included in crystallization and model building. (*b*) A schematic showing the jelly-roll fold. The α3 helix connecting β-strands 2 and 3 is highlighted in yellow.

**Figure 2:**
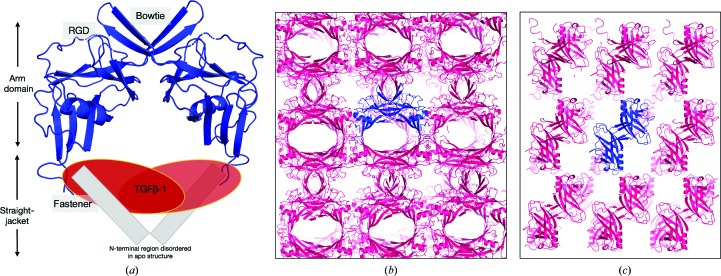
Crystal packing reveals space for the highly dynamic straight-jacket domain. (*a*) Overall structure of the biological dimer. Red ovals represent TGFβ-1 and grey rectangles indicate N-terminal elements that are missing in the apo structure. These regions are positioned to approximate the binding position in the latent complex. The bowtie contains two disulfide bonds that connect the biological dimer. RGD (Arg-Gly-Asp) indicates the integrin-binding motif. (*b*, *c*) Representative crystal-packing diagrams at orientations of 90° relative to one another. For clarity, one dimer is shown in blue.

The structure of unbound (apo) LAP was determined to a resolution of 3.5 Å by X-ray crystallography to understand the conformational changes that occur in LAP during TGFβ-1 binding [PDB entry 6p7j; Fig. 1 ▸(*a*)]. The protein crystallized in space group *C*222 and there is one monomer in the asymmetric unit. Electron density allowed residues 106–126, 129–241 and 244–268 (159 in total) to be modelled, representing most of the protein, with the exception of the straight-jacket domain (residues 30–74) [Fig. 1 ▸(*b*)]. The core architecture of LAP is composed of a jelly-roll β-sandwich fold [two antiparallel, four-stranded β-sheets; Fig. 1(*c*)]. Two adjacent asymmetric units form two inter-chain disulfide bonds (bowtie) to assemble the biological dimer (Fig. 2 ▸). Electron density is missing or weak for the N-terminal straight-jacket domain that binds and cages TGFβ-1 in the latent complex [Fig. 2 ▸(*a*)]. This domain is composed of the α1 helix and latency lasso that have been shown in solution studies to be extended and flexible when unbound to TGFβ-1 (Stachowski *et al.*, 2019 ▸). The solvent content is 45% (Matthews, 1968 ▸) and because the LAP dimer forms a ring-like shape there is space to accommodate movement of this region within the lattice and avoid stabilization by crystal packing [Figs. 2 ▸(*b*) and 2 ▸(*c*)].

**Figure 3:**
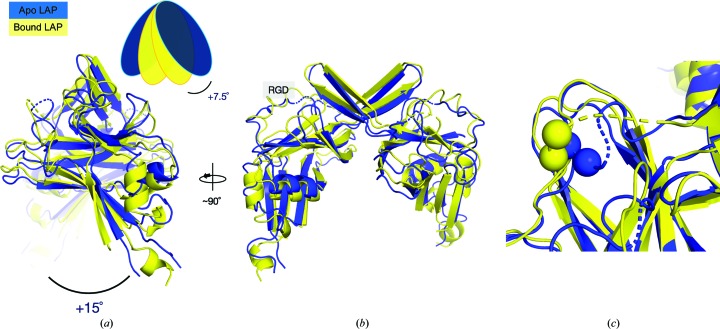
Comparison of apo LAP and TGFβ-1 bound (LTGFβ-1) structures. Only residues modelled in the apo structure were included for comparison. The apo structure reported here (blue; PDB entry 6p7j) is aligned with pig TGFβ-1 bound LAP (yellow; PDB entry 3rjr; Shi *et al.*, 2011 ▸). (*a*) The side view shows that the inter-monomer angle in the apo structure is 15° greater than that in the bound structure. For clarity, the blob diagram approximates this movement of the arm domains. The angle measured here reflects the shift of one monomer in the bound structure relative to the same monomer in the apo structure. (*b*) Front orientation of the alignment. RGD indicates the integrin-binding motif. (*c*) A close-up view of the RGD-containing loop shows that it is similarly positioned in both structures, on the solvent-exposed shoulder of the arm domain. For clarity, the C^α^ atoms of Gly and Asp (which are modelled in both structures) from the motif are shown as spheres.

The crystallization buffers for apo LAP and LTGFβ-1 (Shi *et al.*, 2011 ▸) contained similar amounts of PEG 3350, with sodium as the salt cation, and had pH values (4.6 and 5.6, respectively) far from the p*K*_a_ values of the residues and the isoelectric point of the protein (pI 8.15). These structures were also solved to comparable resolutions (3.5 and 3.05 Å, respectively). The human and pig (from the bound structure) LAP domains share 92% amino-acid sequence identity (Supplementary Fig. S1). However, the structure of apo human LAP solved here is distinct from that of TGFβ-1 bound LAP, with an r.m.s. deviation of 4.032 Å for all 159 C^α^ atoms modelled in the apo monomer. Although the core jelly-roll fold is similar in the absence of TGFβ-1, several structural differences are notable. Firstly, an alignment of the two structures reveals a 15° increase in the angle between monomers in the apo LAP structure (Fig. 3 ▸). This shift was not observed in comparisons of TGFβ-1 bound LAP structures (Zhao *et al.*, 2017 ▸) and suggests that it is unique to LAP in the apo state. This means that in addition to the straight-jacket domain, LAP binding to TGFβ-1 might also include a repositioning of the globular arm domains. This inter-monomer rotation is perhaps accomplished by the residues adjacent to the bowtie functioning as a hinge, ultimately leading to a more ‘open’ conformation of the LAP dimer cavity.

**Figure 4:**
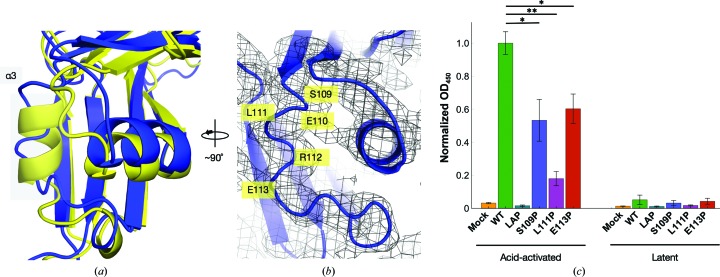
The α3 helix in the latency complex forms during TGFβ-1 binding. (*a*) Alignment of the α3 helix in bound (yellow) and unbound (blue) structures. (*b*) The 2*F*_o_ − *F*_c_ electron-density map of the α3 helix region main chain in apo LAP is shown as a black mesh and is contoured at 1.0σ. (*c*) Normally, TGFβ-1 is only secreted when bound to LAP. To test for the role of the α3 helix in TGFβ-1 binding, HEK293T cells were transfected with proline mutants. After 48 h, the cell-culture supernatant was assayed with an anti-TGFβ-1 antibody, which only recognizes TGFβ-1 when released from LAP. Acid-activated proline mutants formed the LTGFβ-1 complex at much lower levels than wild-type LTGFβ-1. *p < 0.05 and **p < 0.01 (*t*-test).

Finally, the transition to this more ‘open’ conformation appears to require concerted movements distal to the inter-monomer interface and TGFβ-1 binding sites. The main feature of this conformational change is the distortion of the α3 helix in the apo structure that resides on the arm shoulder [Fig. 4 ▸(*a*)]. The density to support main-chain tracing of this region as a loop is well defined [Fig. 4 ▸(*b*)]. There is complete conservation of this region between the pig and human forms, and the region is predicted to be disordered based on the sequence (Supplementary Fig. S1). The importance of the α3 helix in TGFβ-1 binding is supported by experiments measuring the levels of secreted TGFβ-1 with LAP mutants. Normally, TGFβ-1 is dependent on LAP for secretion from cells. To test for latent complex formation, residues in the α3 helix region were mutated to prolines, which prevent the formation of helices (Schulman & Kim, 1996 ▸). The supernatant from transfected HEK293T cells was assayed with an ELISA using an antibody that does not recognize TGFβ-1 when bound to LAP [Fig. 4 ▸(*c*), right]. A short incubation with HCl releases TGFβ-1 (Walton *et al.*, 2010 ▸), allowing it to be detected. LAP mutants showed reduced levels of TGFβ-1 following acid activation [Fig. 4 ▸(*c*), left]. This indicates that lower levels of LTGFβ-1 were secreted and suggests that formation of the α3 helix is important for binding TGFβ-1 and forming the latent complex.

Previous solution studies determined that the straight-jacket region of LAP is flexible and extended when unbound to TGFβ-1; however, the scattering data could not be completely explained by an ensemble of flexible structures sampling conformations of the straight-jacket domain (Stachowski *et al.*, 2019 ▸). This implies that a second structural change occurs in LAP between TGFβ-1 binding states that might explain this discrepancy. This could be a rotation between LAP monomers around the bowtie region, which is suggested by a comparison of the inter-monomer angle between the apo LAP and TGFβ-1 bound LAP crystal structures, but it is unclear whether this this type of conformational change occurs in solution or to what extent. Therefore, to test the contributions of both the straight-jacket domain and a possible bowtie hinge to the observed flexibility of apo LAP in solution, rigid-body modelling was performed using the deglycosylated apo LAP scattering data.

**Figure 5:**
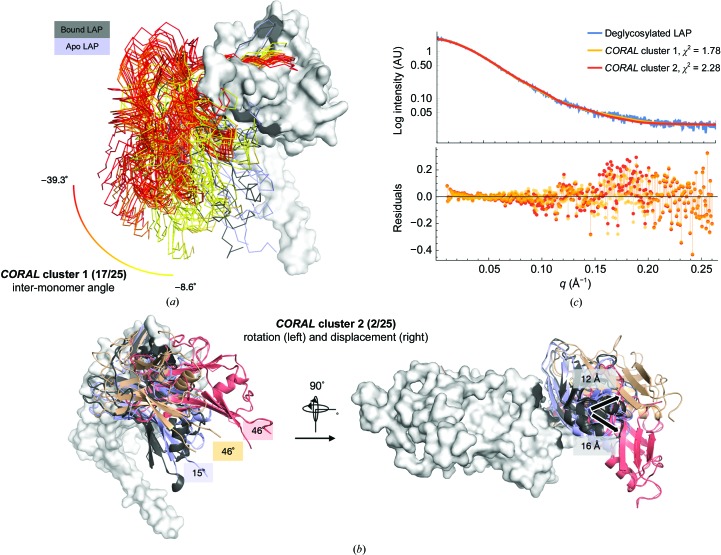
SAXS-based rigid-body modelling and clustering shows that most apo LAP models include a reorientation of the inter-monomer position compared with the TGFβ-1 bound LAP crystal structure. (*a*) *CORAL* models from cluster 1 (gradient from yellow to red illustrating the magnitude of inter-monomer rotation relative to the bound LAP structure) and the apo LAP structure (blue) superimposed onto a single chain of bound LAP (grey/black). For clarity, models are shown without the straight-jacket domain [entire models are shown in Supplementary Fig. S3(*b*)]. (*b*) *CORAL* models from cluster 2 (red and gold) and the apo LAP structure (blue) superimposed onto a single chain of bound LAP (grey/black), indicating rotation (left) and displacement (right) relative to the bound LAP structure. (*c*) Comparison of the average theoretical scattering of models within each cluster with the experimental scattering data (top) with residuals (bottom).

SAXS-constrained rigid-body modelling (*CORAL*) that allowed the sampling of (i) different conformations of the straight-jacket domain (residues 1–75) and (ii) inter-monomer rotations around the bowtie was used to generate models of apo LAP. 25 independent models were generated. Owing to the extensive conformational heterogeneity of the straight-jacket domain, these models were clustered according to the similarity of the arm domains alone (*DAMCLUST*). Clustering analysis yielded two subfamilies with more than one model and is reported in Fig. 5 ▸ and Supplementary Fig. S3. The first cluster is the most populated, with 17 of the 25 models, and the second cluster contains two of the 25 models. The remaining six of the 25 models were clustered individually and therefore can be considered to be outliers (Supplementary Fig. S3). However, all 25 models exhibited inter-monomer rotation. The models in the first cluster contain inter-monomer angles that range from −8 to −39° and are displaced by approximately 3–10 Å [Fig. 5 ▸(*a*) and Supplementary Fig. S3]. The two models in the second cluster are more extended regarding the inter-monomer angle (46.3 and 45.5°) and displacement (16.1 and 12.1 Å) compared with the bound LAP structure and the models in cluster 1 [Fig. 5 ▸(*c*) and Supplementary Fig. S3]. Comparing the experimental scattering with the average theoretical scattering of the models (including the straight-jacket domain) within each cluster yielded χ^2^ values for clusters 1 and 2 of 1.78 and 2.28, respectively [Fig. 5 ▸(*c*)]. The χ^2^ for the average of cluster 1 (1.78) was lower than comparisons with any single *CORAL* model (with the lowest being 1.84; Supplementary Fig. S3). Complete models from cluster 1 are shown in Supplementary Fig. S3.

A visual inspection of an alignment of the models in cluster 1 (gradient from yellow to red) and the apo LAP structure (blue) to the bound LAP structure (black/grey) shows that the bowtie disulfide bonds are maintained and allows the simulation of a hinge [Fig. 5 ▸(*a*)]. Relative to the bound LAP structure, the rotation between the arm domains in cluster 1 and the apo LAP structure is in opposite directions. The former shows rotation leading to a more closed conformation, while the latter shows rotation leading to a more opened conformation. However, several models in the cluster have inter-monomer angles of a similar magnitude to that in the apo LAP structure (Supplementary Fig. S3). Additionally, because most models in this cluster can be superimposed onto the bound LAP reference structure with little displacement (<4 Å), this suggests that different inter-monomer orientations can be accessed by simply rotating perpendicular to the bowtie (Supplementary Fig. S3). This mechanism is also suggested by the apo LAP crystal structure (Fig. 3 ▸). While the average magnitude of the inter-monomer angle of cluster 2 (46°) is similar to several models in cluster 1, the rotation in cluster 2 is accompanied by a large displacement (12–16 Å; Supplementary Fig. S3). The combination of large rotation and large displacement creates a more open conformation of the dimer through both a rotation perpendicular to the bowtie (as in cluster 1 and the apo crystal structure) and a bending along the bowtie [Fig. 5 ▸(*b*)]. This type of orientation is not supported by the crystal lattice. The average theoretical scatterings of the models in each cluster are both in good agreement with the experimental scattering data. The fit for cluster 1 (χ^2^ = 1.78) is slightly improved at mid and high *q* compared with the fit for cluster 2 (χ^2^ = 2.28) [Fig. 5 ▸(*c*)].

Rigid-body modelling shows that the experimental scattering data for apo LAP can be satisfied by individual models that sample conformations of both the inter-monomer angle and the straight-jacket domain (Supplementary Fig. S3). Clustering analysis shows that the majority of these models exhibit similar magnitudes of inter-monomer rotation and displacement that are in agreement with the apo LAP crystal structure, suggesting that this conformation is present in solution. However, previous work showed that apo LAP is best modelled as a mixture owing to the conformational heterogeneity of the straight-jacket domain (Stachowski *et al.*, 2019 ▸). Therefore, to determine (i) whether models with different conformations of both the straight-jacket domain and inter-monomer rotation are preferable to models sampling only the straight-jacket domain and if so (ii) whether multiple inter-monomer rotations are occurring in solution simultaneously, we performed the *Ensemble Optimization Method* (*EOM*; Tria *et al.*, 2015 ▸). To simulate rotation around the bowtie, 10 000 random conformations of the straight-jacket domain were built onto the arm domains of three *CORAL* models with different inter-monomer angles (−26, 47 and 84°), the apo LAP crystal structure (15°) and the bound LAP crystal structure (0°). These models were combined into a single pool of 50 000 models (the Hinge + SJ pool) to use for the genetic algorithm, repeated three times and averaged. This was compared with a run with a pool containing only the 10 000 straight-jacket conformations built around apo LAP (the SJ pool).

**Figure 6:**
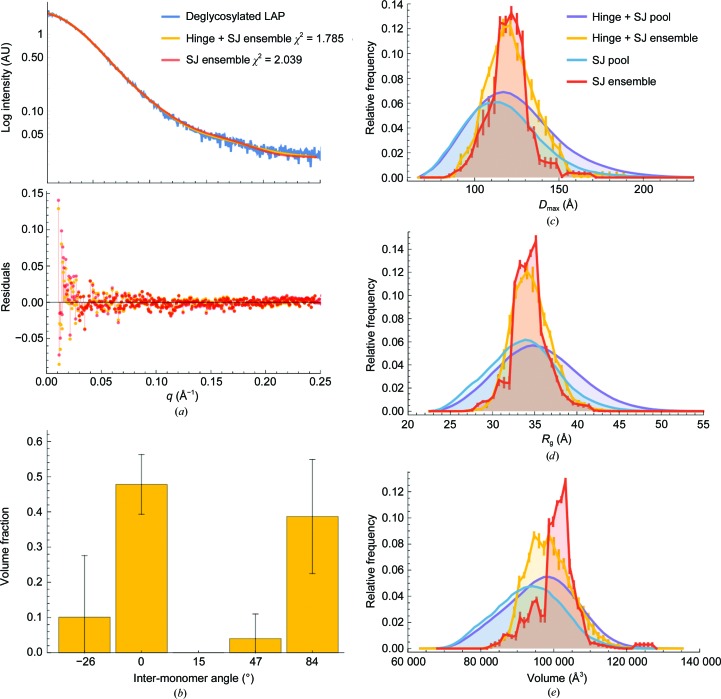
Ensemble modelling suggests that apo LAP in solution consists of a mixture of compact and extended inter-monomer conformations. (*a*) Comparison of theoretical scattering of the ensembles of models with (yellow; Hinge + SJ) or without (red; SJ) inter-monomer rotation around the bowtie hinge and random conformations of the straight-jacket domain. (*b*) Volume fractions of the Hinge + SJ core models determined by the genetic algorithm (*GAJOE*). (*c*, *d*, *e*) Representations of the *D*_max_, *R*_g_ and volume distributions of the random pools and the selected ensembles, respectively. The results reported are the means of three independent runs of the genetic algorithm and error bars represent the standard deviation.

Including inter-monomer rotations around the bowtie with random conformations of the straight-jacket domain improved the fit of the final ensemble to the experimental scattering data from a χ^2^ of 2.04 to 1.785 [Fig. 6 ▸(*a*)]. A visual inspection of the curves and residuals reveals that including the bowtie hinge yielded a slight improvement at low and high *q* [Fig. 6 ▸(*a*)]. The theoretical scattering of the two ensembles are significantly different from one another (*CorMap*; *P* < 10^−6^; Franke *et al.*, 2015 ▸). As expected, including the inter-monomer rotation generated a wider range of conformations in the Hinge + SJ random pool than in the SJ only pool [Figs. 6 ▸(*b*), 6 ▸(*c*) and 6 ▸(*d*)]. However, the radius of gyration (*R*_g_) and maximum distance (*D*_max_) distributions of the Hinge + SJ and SJ only ensembles are almost indistinguishable. Both are well centred compared with their respective random pools, which is characteristic of a globular protein, and have maximum values of approximately 35 and 130 Å for *R*_g_ and *D*_max_, respectively [Figs. 6 ▸(*b*) and 6 ▸(*c*)]. The volume distribution for the SJ only ensemble, however, is more extended than its random pool, suggesting that the protein is extended, which is not consistent with the *R*_g_ and *D*_max_ distributions [Fig. 6 ▸(*c*)]. Even though the volume distribution of the Hinge + SJ random pool is more extended than the SJ only pool, the distribution for the Hinge + SJ ensemble remains centred relative to the random pool, like the *R*_g_ and *D*_max_ distributions [Fig. 6 ▸(*c*)]. The average volume fraction of models in the selected Hinge + SJ ensembles reveals a mainly bimodal distribution of bound (closed) and highly extended inter-monomer orientations [Fig. 6 ▸(*b*)]. The improved fit and internal consistency of the distributions suggests that the Hinge + SJ ensemble explains the experimental scattering data slightly better than the SJ only ensemble. Specifically, because replicates of the genetic algorithm consistently chose two inter-monomer orientations, one of which is a highly rotated conformation of LAP, it suggests that this type of conformation is present in solution. Together with rigid-body modelling, the solution scattering analysis agrees with the mechanism proposed by crystallo­graphic analysis.

### 3.2. Assessing the role of glycosylation in the conformation of LAP using SAXS   

Crystallographic analysis suggests that apo LAP is in a more open conformation than when bound to TGFβ-1. This open conformation is accomplished through inter-monomer rotation and an extended straight-jacket domain. X-ray scattering-based modelling supports this mechanism and suggests that similar conformations occur in solution. However, these studies used deglycosylated LAP because the impact of glycans is notoriously difficult to interpret in structural studies (Guttman *et al.*, 2013 ▸). Importantly, glycosylation can influence protein activity by sterically limiting conformational space (Lee *et al.*, 2015 ▸; Bager *et al.*, 2013 ▸), which may affect the overall folding of LAP since it is heavily glycosylated (Brunner *et al.*, 1992 ▸). The importance of physiological glycosylation on the ability of LAP to sequester TGFβ-1 is undecided (Robertson & Rifkin, 2016 ▸). Therefore, to better assess the role of glycosylation in influencing the folding and flexibility of LAP, SAXS data were collected for apo LAP in three different glycan states: (i) complex (native), (ii) high-mannose and (iii) deglycosylated forms. LAP with high-mannose glycans is produced by expressing the protein in the presence of kifunensine, which is a small-molecule endoplasmic reticulum mannosidase 1 (ERM1) inhibitor. This prevents the complete processing of the glycans from high-mannose to the final complex type and sensitizes the glycans to a commercially available deglycosylase, EndoH [Supplementary Fig. S2(*a*)] (Yu *et al.*, 2011 ▸). Because the high-mannose state is a precursor of the complex form (Doores & Burton, 2010 ▸), comparison of the two is also advantageous for determining whether the folding of LAP changes as the glycans are processed within the cell.

**Figure 7:**
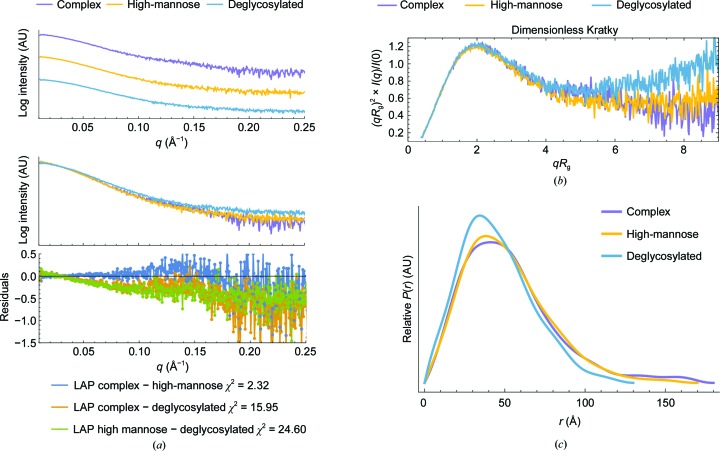
SAXS analysis shows that the flexibility of LAP is independent of glycosylation. The LAP glycoforms analysed are complex (purple), high-mannose (yellow) and deglycosylated (blue) forms. (*a*) Buffer-subtracted scattering curves (top) and comparison of the scattering curves with residuals and χ^2^ values (bottom). (*b*) The dimensionless Kratky plots and (*c*) distributions of interatomic distances, *P*(*r*), indicate that the conformation and flexibility of apo LAP is similar in the three glycoforms.

Prior to SAXS data collection, the melting temperature of LAP glycoforms was measured to determine their relative stability. Glycan modification did not affect the melting temperature of LAP and indicates that the proteins were overall folded similarly prior to X-ray exposure [Supplementary Fig. S2(*b*)]. Comparison of the three LAP glycoforms, as calculated with *DATCMP* (Franke *et al.*, 2017 ▸), showed that the complex and high-mannose forms are in good agreement with each other (χ^2^ = 2.32). However, both the complex and high-mannose forms are in poor agreement with the deglycosylated form, with χ^2^ values of 15.95 and 24.60, respectively [Fig. 7 ▸(*a*)]. The residual plot for the fit between the complex and high-mannose forms show good agreement at low *q* but a slight discrepancy at high *q*, indicating that the proteins match in size and shape but do not share some high-resolution features [Fig. 7 ▸(*a*)]. This is expected and is most likely to reflect that the complex and high-mannose glycans are processed to similar masses but are shaped differently owing to differences in branching and composition. Both the complex and high-mannose forms are in poor agreement with deglycosylated LAP in all *q* ranges [Fig. 7 ▸(*a*)]. At low *q* this is most likely to be owing to differences in molecular weight (determined from the volume of correlation, *V*_C_; Rambo & Tainer, 2013 ▸), where the molecular weight of deglycosylated LAP is 59.1 kDa (the theoretical molecular weight is 58.6 kDa) compared with 87.1 and 87.7 kDa for complex and high-mannose forms, respectively (Supplementary Table S5).

The dimensionless Kratky plot provides a semi-quantitative approach to assessing protein shape that is normalized for differences in particle mass and concentration (Durand *et al.*, 2010 ▸). Although the discrepancies between glycosylated and deglycosylated LAP forms at higher *q* indicate differences in shape [Fig. 7 ▸(*a*)], the Kratky plot suggests that the proteins are folded similarly and exhibit the same degree of flexibility [Fig. 7 ▸(*b*)]. This is because all three glycan forms of LAP exhibit a more gradual intensity decay, with a plateau that is characteristic of a globular protein with partial flexibility [Fig. 7 ▸(*b*)].

The idea that glycosylation does not change the overall fold of LAP is further supported by analysis of the distribution of interatomic distances, *P*(*r*) [Fig. 7 ▸(*c*)]. While the maximum particle dimensions (*D*_max_) for the complex and high-mannose forms are larger than for deglycosylated LAP (Supplementary Table S5), the elongation ratio (ER) is similar for all three glycoforms. The ER is a parameter that describes the asymmetry and noncompactness of a protein based on the *P*(*r*) distribution and is calculated by taking the ratio of the area under the *P*(*r*) function after the *P*(*r*) maximum (*i.e.* the most common distance) to that before the *P*(*r*) maximum (Putnam, 2016 ▸). In this way, symmetric objects such as spheres tend to have values of around 1.0, while elongated shapes such as ellipsoids have much larger values (Putnam, 2016 ▸). The ER values for the complex (1.54), high-mannose (1.77) and deglycosylated (1.74) forms suggest that all of the glycoforms are equally asymmetric. Together, these results show that the observed flexibility and overall folding of apo LAP is not influenced by glycosylation. Additionally, they suggest that the binding mechanism detailed here using crystallography and SAXS-based modelling of the deglycosylated form should also occur in other glycoforms.

## 4. Discussion   

The TGFβ superfamily of secreted growth factor ligands is one of the largest protein families in vertebrates. It comprises over 30 types of proteins, including activins, nodals, bone morphogenic proteins (BMPs) and growth and differentiation factors (GDFs), that regulate diverse developmental and homeostatic processes (Weiss & Attisano, 2013 ▸; Wrana, 2013 ▸). The pro-domains of the TGFβ family differ greatly in orientation and sequence similarity. Some are suggested to interconvert between ‘open-arm’ and ‘closed-arm’ conformations on binding their growth factor ligands (Hinck *et al.*, 2016 ▸; Wang *et al.*, 2016 ▸). For example, in contrast to the ‘crossed-arm’ conformation adopted by LAP complexed with TGFβ, it has been shown that pro-BMP-9 adopts an open-arm conformation in which the α1 helices are positioned away from each other. In this conformation the α1 helices do not contribute to growth-factor binding. However, sequence similarity suggests that pro-BMP-9 is able to form a similar ‘crossed-arm’ conformation to LTGFβ-1 (Hinck *et al.*, 2016 ▸; Mi *et al.*, 2015 ▸).

While such a drastic rearrangement from closed-arm to open-arm in LAP is most likely prohibited by the essentiality of disulfide bonding for dimer formation and the α1 helix of the straight-jacket for TGFβ-1 binding (Walton *et al.*, 2010 ▸; Shi *et al.*, 2011 ▸), the results reported here indicate that LAP is nonetheless structurally distinct in the apo state. This was revealed by comparing the apo LAP structure solved here with the crystal structure of LTGFβ-1. Determining the crystal structure of apo LAP was challenging and was impacted by the limited resolution, low diffraction intensities and the total number of reflections (Table 1 ▸). Phase estimates using a molecular-replacement (MR) procedure of iterative rounds of model building with *Rosetta* and phase solution with *Phaser* (DiMaio *et al.*, 2011 ▸) were critical compared with MR with the unmodified target structure (data not shown). Refining with both *Rosetta* and *Phenix* (DiMaio *et al.*, 2013 ▸) also helped to produce a structure with a geometry that is above average relative to structures at the same resolution (data not shown). However, electron density in the apo LAP structure is missing or weak for the straight-jacket domain. This supports previous solution studies that showed that it is flexible and extended when unbound to TGFβ-1 (Fig. 2 ▸) and might explain the limited resolution of the data. Importantly, the apo LAP structure includes a 15° rotation between monomers that is not present in the bound LAP structure (Fig. 3 ▸). This rotation seems to occur through a hinge near the disulfide-linked bowtie that forms the dimer interface. In addition to the bowtie disulfides, several residues that contribute to the dimer interface in LTGFβ-1 have been implicated in disease. Mutating these residues causes an increase in the amount of constitutively active TGFβ-1 released from cells (Walton *et al.*, 2010 ▸; Shi *et al.*, 2011 ▸). However, a more detailed comparison of the positions of the residues adjacent to the bowtie in the apo LAP and LTGFβ-1 structures is prohibited from analysis here owing to the low resolution.

Crystallographic analysis suggests that the open conformation of the apo structure includes a distortion of the α3 helix that connects the top and bottom strands of the core jelly-roll fold (Fig. 4 ▸). This modelling is supported by circular-dichroism experiments, which showed a reduction in helical content in LAP between TGFβ-1 binding states (Stachowski *et al.*, 2019 ▸; McMahon *et al.*, 1996 ▸). The importance of this region in TGFβ-1 binding states is shown in biochemical experiments, where LAP with proline mutations in the α3 helix region yielded significantly less secreted LTGFβ-1 [Fig. 4 ▸(*c*)]. Jelly-roll folds are composed of eight β-strands arranged into two four-stranded sheets (Richardson, 1981 ▸), and are common in viral proteins (Khayat & Johnson, 2011 ▸) and nuclear proteins such as chaperones (Edlich-Muth *et al.*, 2015 ▸). The loops that connect strands are important for ligand binding (Tunnicliffe *et al.*, 2005 ▸; Huan *et al.*, 2013 ▸; Aik *et al.*, 2012 ▸) and protein–protein interactions (Rudenko *et al.*, 1999 ▸; Cheng & Brooks, 2013 ▸; Stehle *et al.*, 1996 ▸). As such, these loops are dynamic structural features (Huan *et al.*, 2013 ▸) that can control conformational changes (Snyder & Danthi, 2017 ▸; Tunnicliffe *et al.*, 2005 ▸; Belvin *et al.*, 2019 ▸). Morphing between apo and bound LAP structures suggests that the formation of the α3 helix transition might communicate TGFβ-1 binding to the fastener region on the inside of the LAP dimer cavity and cause the arms to contract around TGFβ-1 before finally being stabilized by the straight-jacket (Supplementary Movies S1 and S2).

While the structures of both apo LAP and TGFβ-1 bound LAP (PDB entry 3rjr; Shi *et al.*, 2011 ▸) form extensive and similar lattice contacts with the bowtie region, the inter-arm rotation is only observed in the apo structure. Moreover, this rotation is not observed when comparing the TGFβ-1 bound form when unbound and bound to integrin (PDB entry 5ffo; Dong *et al.*, 2017 ▸), where in the latter the bowtie does not contribute to lattice contacts. While it is possible that lattice contacts are responsible for the specific inter-arm orientation found in the apo LAP crystal structure, the presence of a rotation in itself suggests that the arms are flexible with respect to one another. Rotating between arm domains might serve to regulate the accessibility of the LAP dimer cavity for TGFβ-1 binding, and solution scattering-based modelling supports this. However, it remains unclear whether the LAP arm domains exist in a single open conformation or as an ensemble of states in solution in the absence of TGFβ-1. Rigid-body modelling showed that the experimental SAXS data could be satisfied by many individual models, all of which contained inter-monomer rotations and the majority of which exhibited a hinge-like motion as in the crystal structure (Fig. 5 ▸). However, ensemble modelling persistently yielded a bimodal mixture of bound (closed) and highly extended arm domains from a large pool of random conformations (Fig. 6 ▸). A mixture of open and closed states suggests that the LAP dimer cavity is not always equally accessible, but results from both modelling approaches indicate that the bowtie hinge is a regulatory mechanism for TGFβ-1 binding. However, these results do not explain the existence of simultaneous closed and open states with the α3 helix, which seems to signal the contraction of the inter-monomer cavity and that biochemical studies show is important in forming LTGFβ-1.

Previous biochemical studies showed that apo LAP exhibited increased binding to integrin cell-adhesion proteins compared with the TGFβ-1 bound form (Munger *et al.*, 1998 ▸). It was hypothesized that rearrangement from the TGFβ-1 bound to the unbound form included a repositioning of the RGD (Glu-Gly-Arg) motif that improved access for binding. Additionally, the conformation of the bowtie tail that contains the RGD motif is highly variable in previously reported crystal structures and undergoes a large structural change upon integrin binding (Dong *et al.*, 2017 ▸; Zhao *et al.*, 2017 ▸). However, comparison of the apo and TGFβ-1 bound LAP structures here shows that the bowtie tail is similarly situated in both apo and bound structures, on the solvent-exposed shoulder of the arm domain (Fig. 3 ▸). Therefore, the improved access to this site by integrins might be owing to decreased steric hinderance from the increased flexibility of the straight-jacket domain in the apo state. This is supported by the crystal structure of integrin-bound LTGFβ-1, which revealed an interface between the integrin and the latency lasso of the straight-jacket domain in LAP (Dong *et al.*, 2017 ▸).

The results from this study also show that glycosylation does not alter the overall folding of apo LAP in solution (Fig. 7 ▸ and Supplementary Table S5). This is because all glycoforms tested exhibited the same degree of flexibility [Fig. 7 ▸(*b*)] and similar distance distributions [Fig. 7 ▸(*c*)]. Although in this study the glycans were removed after cellular processing, these results still suggest that dimeric apo LAP can be effectively engineered for expression in simpler systems that do not support post-translational modifications, such as *Escherichia coli*, which could greatly increase the speed of therapeutic development (Harding & Feldman, 2019 ▸; Du *et al.*, 2019 ▸). Additionally, since there were no conformational differences between the high-mannose and complex LAP glycoforms, this suggests that increasing or modifying the branching type of glycosylation will not alter the apo LAP conformation either, which is one approach that has been employed to prolong the circulating half-life of protein therapies and improve their overall pharmacokinetic profiles (Perlman *et al.*, 2003 ▸; Keck *et al.*, 2008 ▸). Although these results improve the therapeutic potential of recombinant LAP, they do not provide an answer to why deglycosylation induces the dissociation of TGFβ-1 from LAP (Miyazono *et al.*, 1992 ▸; Robertson & Rifkin, 2016 ▸) while glycan modification does not alter the folding of LAP or its ability to bind TGFβ-1 (McMahon *et al.*, 1996 ▸). This seems to suggest that undetermined structural differences remain between apo and TGFβ-1 bound LAP, where the orientation of bound LAP is susceptible to a deglycosylation-induced conformational change into the apo LAP state.

While these results reveal new spatial details regarding the TGFβ-1 binding mechanism during LTGFβ-1 processing and sequestration by recombinant LAP, they cannot definitively explain the temporal sequence of conformational changes or explain whether similar rearrangements occur during TGFβ-1 release from LAP and when LAP is tethered to the extracellular matrix (Liénart *et al.*, 2018 ▸). The binding and release pathways are suggested to include different structural changes and the release pathway itself is different depending on the perturbation (Jobling *et al.*, 2006 ▸). These problems are challenging and might not be resolvable with SAXS or crystallo­graphy alone, and the molecular weight of LAP makes it a difficult target for structural studies using NMR or cryo-EM. In summary, the combination of X-ray crystallography, SAXS and biochemical analyses has provided novel insights into the required conformational changes in LAP for TGFβ-1 binding that can potentially aid in the development of therapeutics targeting and use of LAP to modulate the activity of TGFβ-1.

The deglycosylated LAP SAXS data used for modelling are available in the supporting information. These data have been deposited in SASBDB (the Small Angle Scattering Biological Data Bank; Valentini *et al.*, 2015 ▸) as entry SASDFD2.

## 5. Related literature   

The following references are cited in the supporting information for this article: Drozdetskiy *et al.* (2015 ▸) and Gasteiger *et al.* (2005 ▸).

## Supplementary Material

PDB reference: latency-associated peptide, 6p7j

SASBDB reference: wild-type human latent transforming growth factor β-1 (LTGFB-1), SASDFD2

Supplementary tables and figures. DOI: 10.1107/S205225251901707X/mf5037sup1.pdf

Supplementary movie S1: front view of a morph between apo and bound LAP structures. DOI: 10.1107/S205225251901707X/mf5037sup2.mov

Supplementary movie S2: side view of a morph between apo and bound LAP structures. DOI: 10.1107/S205225251901707X/mf5037sup3.mov

## Figures and Tables

**Table 1:** Data-collection and refinement statistics Values in parentheses are for the outermost shell.

Data collection	
Diffraction source	IMCA-CAT, APS, ANL
Detector	PILATUS 6M
Temperature (K)	100
Wavelength (Å)	1.0
Rotation range per image (°)	0.25
Total rotation range (°)	137.5
Reflections (measured/unique)	6476/3328
Space group	*C*222
*a*, *b*, *c* (Å)	51.06, 154.9, 62.25
α, β, γ (°)	90, 90, 90
Resolution (Å)	36.31–3.50 (3.63–3.50)
*R*_p.i.m._	0.143 (1.314)
〈*I*/σ(*I*)〉	3.4 (0.91)†
CC_1/2_	0.948 (0.248)
Completeness (%)	99.6 (98.5)
Multiplicity	1.9 (1.9)
Refinement	
Resolution (Å)	36.31–3.50 (3.63–3.50)
*R*_work_/*R*_free_	0.288/0.321 (0.329/0.331)
Reflections in working set	3169 (324)
Reflections in test set	159 (12)
Total No. of atoms	1298
Average *B* factor (Å^2^)	111.1
R.m.s. deviations	
Bond lengths (Å)	0.003
Bond angles (°)	0.736
Ramachandran plot	
Favoured (%)	85.62
Allowed (%)	14.38
Outliers (%)	0
Molecules in asymmetric unit	1
PDB code	6p7j

† The mean *I*/σ(*I*) falls below 2.0 at 4 Å.
