# Supplementary material for: Structural insights into conformational switching in latency-associated peptide between transforming growth factor β-1 bound and unbound states
Source: IUCrJ. 2020 Feb 6;7(Pt 2):238–52. doi: 10.1107/S205225251901707X (PMC7055372; doi:10.1107/S205225251901707X)
Supplement: Supplementary file 1 [file m-07-00238-sup1.pdf]

IUCrJ

Volume 7 (2020)

Supporting information for article:

Structural insights into conformational switching in latency-associated peptide between TGFβ-1-bound and unbound states

Timothy R. Stachowski, Mary E. Snell and Edward H. Snell

Table S1 Macromolecule production information

Source organism	*Homo sapiens*
Forward primer	CATCACCATCACCATCACTGACACCACCATCAC
Reverse primer	CCTTCTGTGCCGGCTGCTCTGCAGATG
Cloning vector	pTT3
Expression vector	pTT3
Expression host	HEK293
Complete amino acid sequence of the construct produced	LSTsKTIDMELVKRKRIEAIRGQILSKLRLASPPSQGEVPPGPLPEAVLALYNSTR DRVAGESAEPEPEPEADYYAKEVTRVLMVETHNEIYDKFKQSTHSIYMFFNTSE LREAVPEPVLLSRAELRLLRLKLKVEQHVELYQKYSNNSWRYLSNRLLAPSDSP EWLSFDVTGVVRQWLSRGGEIEGFRLSAHCSCDSRDNTLQVDINGFTTGRRGD LATIHGMNRPFLLLMATPLERAQHLQSSRHRaHHHHHH

The underlined sequence is the C-terminal His6 tag.

Lowercase letters signify mutations from native sequence.

Table S2 Crystallization

Method	microbatch under oil
Plate type	72-well Terasaki (Sigma)
Temperature (K)	273
Protein concentration	4 mg ml^{-1}
Buffer composition of the protein solution	137 mM NaCl, 2.7 mM KCl, 10 mM Na_2HPO_4, 1.8 mM KH_2PO_4
Composition of reservoir solution	10% PEG 3350, 300 mM NaAc pH 4.6
Volume and ratio of drop	2 uL, 1:1

Table S3 SAXS data collection parameters

Instrument	Advanced Light Source SIBYLS SAXS Beamline with 2M detector (Dyer *et al*., 2014)
Wavelength (Å)	1.27
Camera length (m)	2
q measurement (Å^{-1})	0.01087 – 0.2610
Monitoring for radiation damage	data frame-by-frame comparison, CorMap (Franke *et al*., 2017)
Sample configuration	static sample cell
Sample temperature (˚C)	10

Table S4 Software employed for SAXS data reduction, analysis, and interpretation

Data reduction	Solvent subtraction using PRIMUSqt (ATSAS 2.8.3 (Franke *et al*., 2017)
Extinction coefficient	ProtParam (Gasteiger *et al*., 2005)
Basic analyses	PRIMUSqt (ATSAS 2.8.3 (Franke *et al*., 2017))
Atomic structure modelling	CRYSOL 2.8.3 (Svergun *et al*., 1995)
Missing sequence modelling	MODELLER via CHIMERA (Yang *et al*., 2012)
Rigid body modelling	CORAL 1.1 (Petoukhov *et al*., 2012)
Model clustering	DAMCLUST (Petoukhov *et al*., 2012)
Ensemble modelling	EOM 2.1 (Tria *et al*., 2015)

Table S5 Experimental determined parameters from SAXS analysis

Sample	LAP native		LAP HM		deglyLAP	
Concentration (mg ml^{-1})	1.0	0.7	1.0	0.7	1.4	0.7
Guinier Analysis						
I(0) (arbitrary units)	2.14 +/- 0.012	3.10 +/- 0.022	2.16 +/- 0.017	2.04 +/- 0.018	1.99 +/- 0.01	1.56 +/- 0.01
Rg (Å)	38.79 +/- 0.83	40.22 +/- 1.77	39.63 +/- 1.81	42.46 +/- 1.93	35.09 +/- 1.09	36.44 +/- 1.22
q min (Å^{-1})	0.0125	0.0164	0.0125	0.0119	0.0136	0.0136
qRg max	1.29	1.22	1.20	1.27	1.28	1.29
P(r) analysis						
I(0) (arbitrary units)	2.21 +/- 0.016	3.19 +/- 0.021	2.18 +/- .0145	2.08 +/- 0.016	1.99 +/- 0.006	1.61 +/- 0.015
Rg (Å)	42.67 +/- 0.65	43.7 +/- 0.582	40.9 +/- 0.501	45.68 +/- 0.639	35.53 +/- 0.191	40.07 +/- 0.827
D_{max} (Å)	180	190	175	190	130	170
q range (Å^{-1})	0.0125-0.2611	0.0164-0.2611	0.0125-0.2611	0.0119-0.2611	0.0136-0.2611	0.0136-0.2611
Porod volume (Å^{-3})	164000	165000	174000	183000	129000	123000

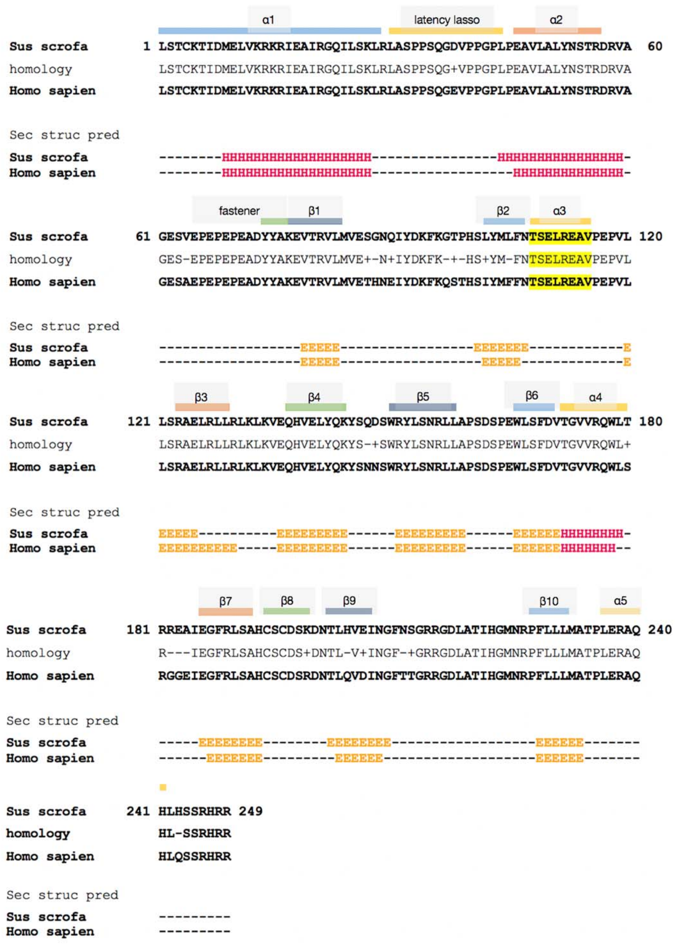

Figure S1 Alignment of human and pig LAP sequences and secondary structure prediction. Alignment between the two sequences shows 92% conservation. The α3-helical region is highlighted in yellow. Secondary structure prediction was performed with JPred4 (Drozdetskiy *et al.*, 2015).

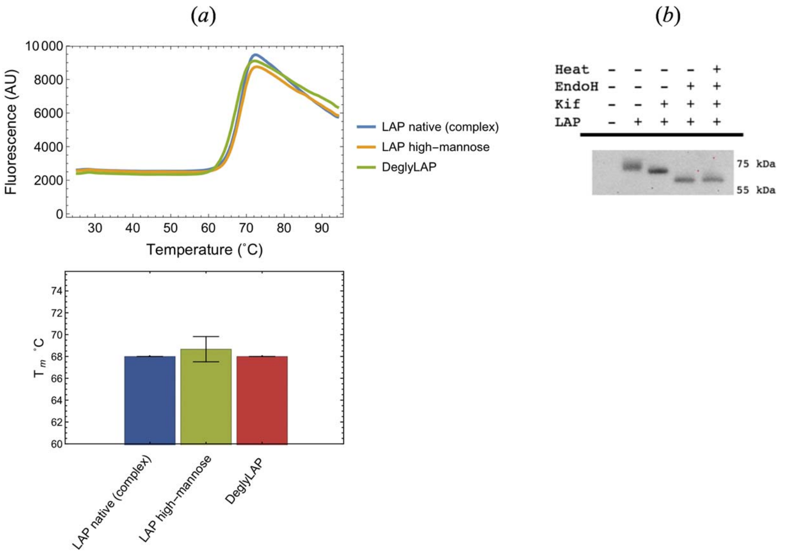

Figure S2 (a) Differential scanning fluorimetry measurements show that modification of glycosylation does not change the LAP melting temperature. (top) Shows a representative melting curve. (bottom) Melting temperature was determined with taking the maximum value from the first derivative of the fluorescence intensity curve. Experiments were repeated in triplicate and reported as the average across measurements +/- the standard deviation. (b) Western blot of HEK293 supernatants showing the decrease in molecular weight with enzymatic digestion of glycans. LAP was probed with Anti-TGFβ-1 (cat. no. 3711, Cell Signaling Technology).

(*a*)

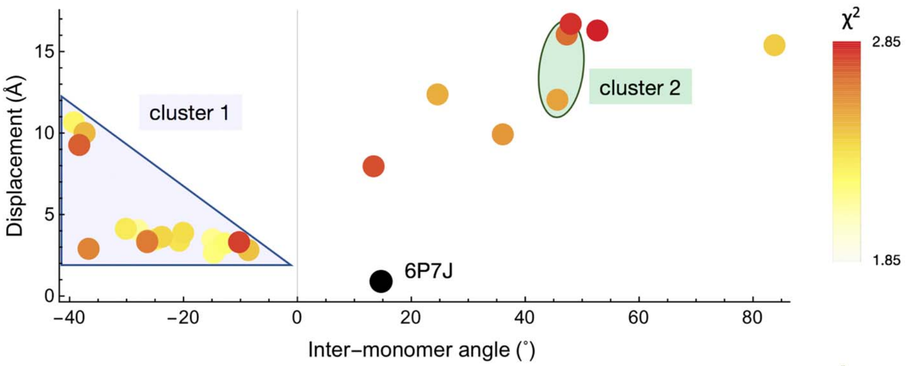

(*b*)

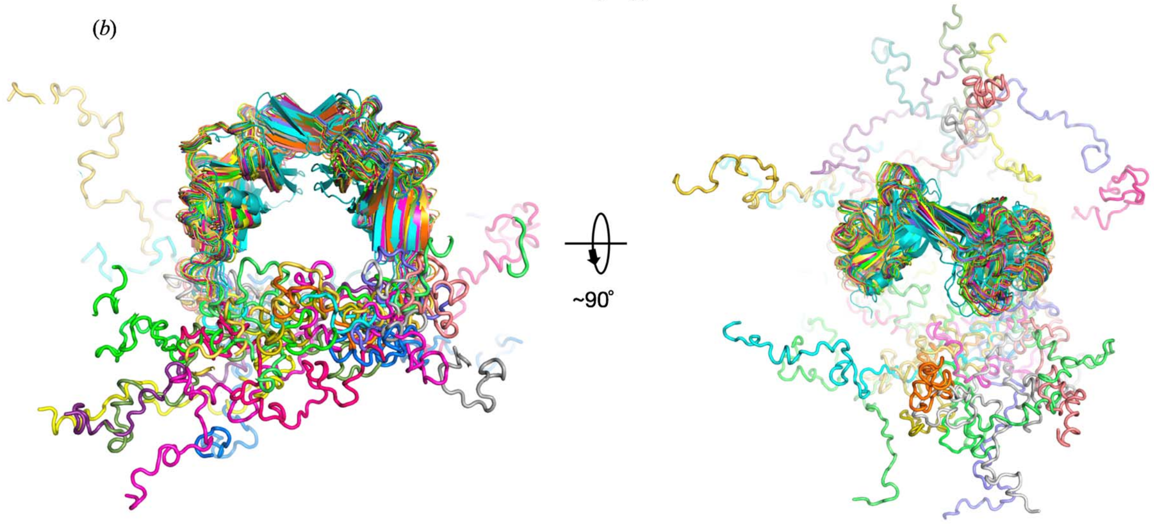

Figure S3 Models from SAXS-based rigid body modelling (CORAL). (a) Plot of the displacement and inter-monomer angle (as calculated with PyMOL) for each model and apo LAP structure (black) relative the the bound LAP structure (PDB 3RJR). Red-yellow colouring for χ^2 values indicate the goodness of fit between the theoretical scattering of the entire model to the experimental SAXS data. Clustering with DATCLUST (blue and green) was performed with only the arm domains. (b) Aligned models from cluster 1.
